# Inability to obtain sperm for fresh IVF cycles: analysis and incidence of outcomes using a database from the United States

**DOI:** 10.1186/s40738-020-00082-3

**Published:** 2020-08-11

**Authors:** Alexandra Joice Berger, Valary Raup, Ramy Abou Ghayda, Andrea Lanes, Martin Kathrins

**Affiliations:** 1 Division of Urology, Brigham and Women’s Hospital and Harvard Medical School, 45 Francis St ASB-II, Boston, MA 02115 USA; 2 Department of Obstetrics and Gynecology, Center for Infertility and Reproductive Surgery, Brigham and Women’s Hospital and Harvard Medical School, 75 Francis Street, Boston, MA 02115 USA

## Abstract

**Background:**

Azoospermia is present in 10% of men presenting with infertility and surgical sperm retrieval rates for men with azoospermia due to spermatogenic dysfunction remain low. We investigated the incidence of failed fresh IVF cycles due to inability to obtain sperm and describe predictors for subsequent IVF.

**Methods:**

A national IVF database was used to identify fresh IVF cycles in which there was failure to obtain sperm. Patient linkage was utilized to determine outcomes of subsequent IVF.

**Results:**

243,291 fresh IVF cycles were identified; 719 (0.3%) listed “inability to obtain sperm” as reason for embryo non-transfer. Male infertility was a factor in 537 (75%) and ejaculation was the most common anticipated sperm source (414, 57%). 713 (99.2%) cycles resulted in retrieved oocytes, but only 627 (87.2%) cryopreserved oocytes. 265 (37%) of couples underwent subsequent IVF. On multivariable analysis, lack of initial oocyte cryopreservation (OR 0.34, *p* = 0.01) and male infertility (OR 0.14, p = 0.01) were associated with having no subsequent cycles. Partner sperm was used in 213 (80%) second cycles and sperm retrieval method was largely conserved (181/213, 85%). Embryos were transferred in 186 (70%) second cycles. Failed embryo transfers were due to repeat inability to obtain sperm in 5 (6%) cycles.

**Conclusions:**

Failure to obtain sperm during fresh IVF is rare, but most affected couples will not pursue further cycles of IVF after their initial failed attempt.

## Introduction

Azoospermia is present in 3–10% of men presenting with infertility [4, 16]. Unfortunately, surgical sperm retrieval (SSR) rates for men with nonobstructive azoospermia (NOA) remain low [8]. Furthermore, other reasons for failure to obtain sperm on the day of in-vitro fertilization (IVF) include ejaculatory dysfunction and transient/unexpected azoospermia. Failure to obtain sperm for assisted reproductive therapies is a significant stressor for couples already suffering from infertility.

Multiple different approaches to SSR have been pursued to optimize outcomes. A “fresh” SSR attempt involves timing the man’s extraction procedure to a programmed ovulation induction cycle and oocyte retrieval. Failure to obtain sperm in such a scenario is particularly devastating for the couple. An alternative approach involves elective SSR with cryopreservation of sperm—if successful—and use of the thawed specimen later with in-vitro fertilization (IVF) [1]. However, there are technical limitations of sperm cryopreservation in this setting including rare occurrences of complete post-thaw cellular loss [6]. Thus, there is no consensus on the optimal approach.

While individual centers may report outcomes regarding canceled cycles of IVF due to inability to obtain sperm, there is no multi-institutional, national data on the real-world incidence. Furthermore, little is known about the clinical follow-up for such couples regarding their decision to pursue future cycles of IVF. Beyond failed SSR in the setting of azoospermia, there are yet other less common reasons why sperm may not be available—such as failure to obtain sperm from a planned ejaculated specimen—for which no incidence data exists. Herein, we sought to investigate the incidence and clinical outcomes for couples whose fresh IVF cycles were canceled due to an inability to obtain sperm.

## Materials and methods

Exemption was obtained from Institutional Review Board at Brigham and Women’s Hospital for this study. A retrospective analysis was performed of the Society for Assisted Reproductive Technology (SART) Clinical Outcome Report System database of all fresh IVF cycles for which there was a failure to obtain sperm. SART is an affiliate society of the American Society of Reproductive Medicine and is a national consortium of assisted reproductive technology centers in the U.S. Relevant data was available from 2014 to 2016. We analyzed couples’ subsequent linked cycles of IVF after their initial failed cycles. Demographic data included region of IVF center, ethnicity of male and female partner, obstetric history, reason for IVF (multiple reasons possible), intended sperm source (ejaculation, epididymal aspirate, testicular extraction, electroejaculation, retrograde ejaculation). Cycles involving a gestational carrier were excluded from our analysis. Outcomes regarding female partner included number of oocytes retrieved and the decision to cryopreserve oocytes.

The statistical analyses were performed utilizing Stata 14 (College Station, TX: StataCorp LP). Chi squared tests and logistic regression analyses were used. A *p*-value of < 0.05 was regarded as statistically significant and all analyses were two-tailed. Results are presented as odds ratios (OR).

## Results

243,291 total fresh IVF cycles were identified from 2014 to 2016. Amongst these, 719 cycles (including 710 couples) listed “inability to obtain sperm” as the reason for cycle failure, with an annual incidence of 0.3% (range 0.2–0.4%) (Table 1).

**Table 1:** Yearly incidence of sperm “no retrieval” cycles

	Number of cycles with failure to obtain sperm	Total number of fresh IVF cycles	Percentage
Reporting year			
2014	173	85,572	0.2%
2015	258	81,712	0.3%
2016	288	76,007	0.4%
Overall	719	243,291	0.3%

The patient demographics for these cycles are included in Table 2. The largest group of patients were White and from the Northeast. Most had no prior pregnancies and had never pursued prior IVF cycles. Male infertility was the reason for IVF in only 537 (75%) cycles. Ejaculation was the most common anticipated sperm source, followed by testicular biopsy and epididymal aspirate.

**Table 2:** Sperm “no retrieval” cycle demographics. Total patients = 719

	Number of cycles with failure to obtain sperm	
Reporting year		
2014	173	24.1%
2015	258	35.9%
2016	288	40.1%
Clinic region		
Northeast	290	40.3%
South	242	33.7%
West	95	13.2%
Midwest	92	12.8%
Race		
White	216	30.0%
Black	70	9.7%
Hispanic	28	3.9%
Asian	55	7.6%
Native American	1	0.1%
Unknown	351	48.8%
Gravidity		
0	447	62.2%
1 to 2	199	27.7%
3+	71	9.9%
Unknown	2	0.3%
Prior fresh cycles		
0	504	70.1%
1 to 2	178	24.8%
3+	37	5.1%
Reason for infertility (more than one is possible)		
Male infertility	537	74.7%
Endometriosis	24	3.3%
PCOS	64	8.9%
Diminished ovarian reserve	143	19.9%
Tubal issues	63	8.8%
Uterine	39	5.4%
Unexplained	32	4.5%
Complication		
No	712	99.0%
Yes	7	1.0%
Infection	1	0.1%
Hyperstimulation	4	0.6%
Other	3	0.4%
Sperm source		
Ejaculation	414	57.6%
Testicular	251	34.9%
Epidiymal	50	7.0%
Retrograde ejaculation	1	0.1%
Electro-ejaculation	1	0.1%
Unknown	2	0.3%
Oocytes retrieved		
No	6	0.8%
Yes	713	99.2%
Oocytes frozen		
No	92	12.8%
Yes	627	87.2%

Most (99.2%) cycles resulted in retrieved oocytes, but oocytes were cryopreserved in only 87.2%. On univariate analyses, smoking (vs nonsmoking) as well as reporting years 2015 and 2016 (vs 2014) were associated with cryopreservation. On multivariable analysis, however, only reporting year 2015 and location in the Northeast, Midwest, and West (vs South) were associated with oocyte cryopreservation (Table 3).

**Table 3:** Multivariable analysis of factors predicting cryopreservation of eggs

	Odds Ratio	p-value	[95% Conf. Interval]
Female partner active smoker	1.92	0.22	0.68–5.47
Female partner age	1.06	0.074	0.99–1.14
Male infertility diagnosis	1.11	0.809	0.48–2.59
Non-white race	1.10	0.804	0.53–2.29
Reporting Year			
2014	reference		
**2015**	**3.08**	**0.013**	**1.27–7.49**
2016	1.69	0.284	0.65–4.41
Region			
South	reference		
**Northeast**	**3.32**	**0.004**	**1.47–7.48**
**Midwest**	**3.40**	**0.033**	**1.1–10.44**
**West**	**5.06**	**0.005**	**1.64–15.58**

**Fig. 1:**
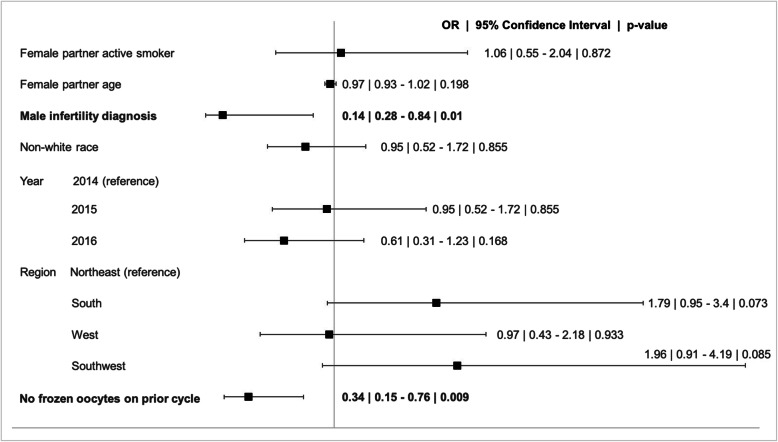
Forest plot demonstrating multivariable analysis of predictors of undergoing subsequent cycles

265 (37%) of couples underwent subsequent IVF cycles. On multivariable analysis, lack of cryopreservation of oocytes on initial cycle and an initial diagnosis of male infertility were associated with failure to undergo subsequent cycles (Table 4 and Fig. 1).

**Table 4:** Multivariable analysis of predictors of undergoing subsequent cycles

	Odds Ratio	p-value	[95% Conf. Interval]	
Female partner active smoker	1.06	0.87	0.55	2.04
Female partner age	0.97	0.20	0.93	1.02
**Male infertility diagnosis**	**0.14**	**0.01**	**0.28**	**0.84**
Non-white race	0.78	0.30	0.48	1.25
Reporting Year				
2014	reference			
2015	0.95	0.86	0.52	1.72
2016	0.61	0.17	0.31	1.23
Region				
Northeast	reference			
South	1.79	0.07	0.95	3.40
West	0.97	0.93	0.43	2.18
Southwest	1.96	0.09	0.91	4.19
**No frozen oocytes on prior cycle**	0.34	**0.01**	**0.15**	**0.76**

Donor sperm was used in 52 (19%) second IVF cycles. Of the couples who used partner’s sperm, the method of sperm retrieval was largely conserved from the first IVF attempt (181/213, 85%). Embryos were transferred in 186 (70%) of second cycles, with a clinical pregnancy rate of 34% (89/265) and a live birth rate of 28% (73/265). Failed embryo transfers during second IVF cycle were due to repeat inability to obtain sperm (5, 6.4%), oocyte/embryologic reasons (52, 65.8%) and other reasons (13, 16.5%).

## Discussion

IVF cycles which are canceled due to an inability to obtain sperm are rare, occurring only in 0.3% of cycles (1 in 338 cycles). Here, we report the first real-world incidence of such instances based on a national cohort. Most of these couples planned to use ejaculated sperm for IVF, followed by planned use of testicular sperm. We also observed that a minority of couples attempted a subsequent cycle of IVF, with most couples utilizing the same planned sperm source.

Inability to obtain sperm is the most feared outcome for an azoospermic man undergoing planned SSR in conjunction with programmed ovulation induction, or “fresh” testicular sperm extraction (TESE). For men with azoospermia due to spermatogenic dysfunction, also known as non-obstructive azoospermia, sperm retrieval rates (SRR) remain relatively low. A recent meta-analysis showed successful retrieval occurs in only 52% of surgeries when microsurgical testicular sperm dissection is performed [2]. However, for “fresh” TESE, availability of an operating microscope may be limited as may operating room availability, so a conventional non-microsurgical TESE must be carried out. For that latter procedure, successful retrieval rates are lower.

For men with obstructive azoospermia (OA), such as those who have undergone prior vasectomy, SRR should be practically 100% [3]. Even if initial percutaneous testicular or epididymal aspiration attempts are unsuccessful, a “back-up” approach employing conventional TESE will almost always be successful. Interestingly, we observed that epididymal aspirate was the planned sperm in 7% of such failed cycles. It is unclear why these couples did not undergo same-day open TESE after aspiration failed to obtain sperm. However, while nomogram predictions, reliant on testicular size and serum FSH, are highly effective at differentiating between men with NOA and idiopathic OA, they are not perfectly accurate [13]. Furthermore, men with underlying diffuse maturation arrest testicular histology may have larger testicles and relatively lower FSH, relative to other men with NOA [17], and may be mistakenly planned for an epididymal aspirate. Thus, scenarios in which a “fresh” TESE is planned with the assumption of a high retrieval rate due to presumed obstructive physiology, may in fact result in failure to obtain sperm due to unexpected spermatogenic dysfunction.

Our results are surprising insofar as most of the instances of inability to obtain sperm for IVF relied on ejaculated sperm, indicating varied causes including sexual dysfunction or insufficient numbers of ejaculated sperm. Delayed orgasm or anorgasmia, however transient, may ultimately be at fault. Secondary orgasm dysfunction, resulting later in life, may be due to selective serotonin reuptake inhibitors, hyperprolactinemia, chronic penile stimulation, or psychogenic/situational reasons [5]. This result underscores the importance of a sexual history intake during the couple’s initial evaluation for infertility. Abnormal findings should prompt referral to a male reproductive medicine specialist [11]. Options for such men may include cryopreservation of ejaculated sperm ahead of time, planned electroejaculation in cases of known ejaculatory failure, penile vibratory stimulation, or even SSR. Electroejaculation has a high success rate among men with psychogenic anorgasmia, but requires sedation [12]. One study found the risk of transient azoospermia on the day of IVF is 52% among men with a prior semen analysis with a total count less than 100,000. Thus, such men with cryptozoospermia or severe oligozoospermia are at high risk for transient azoospermia and should be especially encouraged to cryopreserve sperm [7].

Sperm cryopreservation may help avoid instances of failed IVF cycles due to an inability to obtain sperm. Cost for elective sperm cryopreservation remain high and insurance coverage in men without azoospermia is poor. Unfortunately, there is limited insurance coverage for sperm cryopreservation and out-of-pocket costs can be significant at over $1000 for processing, with further yearly fees for maintenance [15]. The fertilization and pregnancy rates are similar when comparing “fresh” versus cryopreserved/thawed testicular sperm obtained from men with NOA [9]. However, even prior sperm cryopreservation may not guarantee the presence of sperm for IVF as there are rare instances of post-thaw cellular loss among men with severe oligozoospermia or cryptozoospermia [6]. Yet, such costs of sperm cryopreservation pale in comparison to the costs of a failed IVF cycle due to the unavailability of sperm.

The costs and medical risks of IVF are a concern, making it of paramount importance to avoid such instances of canceled IVF cycles. While insurance mandates for IVF coverage are expanding, in the absence of such coverage, out-of-pocket costs for IVF can exceed $20,000 [18]. The risks of ovulation induction include rare instances of ovarian hyperstimulation syndrome and surgical risk, which otherwise would be avoided if IVF were forgone due to a prior knowledge of an inability to obtain sperm [14]. Furthermore, one study found that only a minority of women would ultimately opt to use donor sperm after suffering a failed cycle of IVF due to an inability to surgically obtain sperm from her partner [10]. Indeed, this attitude was confirmed in our study, as only a minority of couples attempted a further cycle of IVF after the initial failure with even fewer subsequently utilizing donor sperm.

While this is a large national cohort, one limitation includes the retrospective nature of the data. There is limited granularity regarding the underlying etiology of male factor infertility (e.g., obstructive azoospermia due to vasectomy versus spermatogenic dysfunction), which precludes further the generalizability of the results. The database does not allow for linking to previous semen analyses prior to IVF. Furthermore, the database utilized only includes data from 2014 to 2016 and there were only 719 total cycles in which there was failure to obtain sperm. As data from subsequent years becomes available additional conclusions may be drawn with more representative cycles. A future area of potential study is comparing this data from the United States with data obtained within similar databases in other countries. Furthermore, there is a need for future multi-intuitional cohorts to examine this question with more detail about the male partners history.

## Conclusion

This study shows that IVF cycles are only rarely canceled to an inability to obtain sperm. Ejaculated sperm was the most common expected source of sperm leading to cancelation. Most affected couples will not pursue further cycles of IVF after their initial failed attempt.

## Data Availability

The datasets during and/or analyzed during the current study available from the corresponding author on reasonable request.
